# Virtual Screening of Molecules via Neural Fingerprint-based Deep Learning Technique

**DOI:** 10.21203/rs.3.rs-4355625/v1

**Published:** 2024-05-09

**Authors:** Rivaaj Monsia, Sudeep Bhattacharyya

**Affiliations:** University of Wisconsin- Eau Claire; University of Wisconsin- Eau Claire

**Keywords:** Artificial neural network, deep learning, drug design, machine learning, neural network-based fingerprinting, structure-based inhibitor design

## Abstract

A machine learning-based drug screening technique has been developed and optimized using convolutional neural network-derived fingerprints. The optimization of weights in the neural network-based fingerprinting technique was compared with fixed Morgan fingerprints in regard to binary classification on drug-target binding affinity. The assessment was carried out using six different target proteins using randomly chosen small molecules from the ZINC15 database for training. This new architecture proved to be more efficient in screening molecules that less favorably bind to specific targets and retaining molecules that favorably bind to it.

**Scientific contribution:**

We have developed a new neural fingerprint-based screening model that has a significant ability to capture hits. Despite using a smaller dataset, this model is capable of mapping chemical space similar to other contemporary algorithms designed for molecular screening. The novelty of the present algorithm lies in the speed with which the models are trained and tuned before testing its predictive capabilities and hence is a significant step forward in the field of machine learning-embedded computational drug discovery.

## Introduction

Computational chemistry is essential to probing complex chemical systems and plays a critical role in computer-aided drug design [1, 2]. With the availability of large datasets, machine learning and artificial intelligence-embedded computational chemistry tools are becoming increasingly popular [3–5] and holds great promise to shed light on several challenging fields of chemistry including structure-guided drug design [2, 6–12]. Drug discovery is a multi-step process; several estimates characterize this process as taking, on average, 12–18 years and around 2.6 billion US$ for a drug to progress from a laboratory discovery to a patient’s bedside. Novel machine learning applications are being explored in expediting certain parts of the drug design timeline, namely compound screening [2, 6–17]. This multidisciplinary research area dominated by computational chemistry, data science, and machine learning seems to hold large untapped resources for structure-guided drug discovery that could alter the landscape of healthcare [6, 11, 13].

In our lab, computational chemistry has been used for over a decade in elucidating the chemistry of enzymes that are drug targets[18–27]. Using classical and quantum physics-based models, these studies explored intermolecular interactions[18, 20, 22]. receptor-ligand complexes[19–21], redox processes[23–26], and enzyme catalysis[19, 21, 23]. However, these physics-based approaches failed to capture the information of selective molecular recognition and translate that to a rigorous inhibitor search. This is because a study of selective inhibition requires a molecular docking process that necessitates the representation of a part of the enzyme’s active site and each small molecule in a database and hence is computationally expensive. Consequently, they were not very effective in molecular screening of a large-molecular database[28]. This prompted us to explore artificial intelligence-embedded computational screening techniques.

A single molecule can be thought of as a sentence composed of atoms or substructures as words [29]. These substructures are combined in different patterns generating various molecular structures. Thus, encoders mimicking those used in natural language processing appear to hold great promise in deciphering molecular substructures that could elucidate molecular recognition by an enzyme. In particular, many such efforts have focused on abstract features of molecules via self-supervised learning, which is then used to train for predicting specific properties [29–33]. In the present study, we integrated a neural fingerprint-based deep learning technique with computer-aided drug design. The new architecture is found to be more efficient in screening molecules to accelerate the process of compound screening.

## Theory and Methods

Computations were carried out on the hybrid GPU-CPU Cluster with 61 nodes and 3904 cores at the Blugold Center for High-Performance Computing, UW-Eau Claire. Proteins were downloaded from Protein Data Bank [34]. VMD was used for visualization and molecular editing [35]. A random set of 50000 molecules was obtained from ZINC15 database [36–38]. For computing molecular properties, the Open Babel codes [39] were used to interchange molecular structures in various formats. Molecular docking on specific target proteins was carried out using AutoDockFR [40]. Reading and writing of machine-readable molecular substructures were done using RDKit [41]. The similarity maps of molecules were generated using Random Decision Forest [42] following the published method [43]. The precision, recall, and receiver operating characteristics were calculated using the scikit-learn (version 1.4) library [44]. The technical implementation of neural network architecture was done in PyTorch [45].

### Preparation of molecular library

ZINC15 stores the structural information of a large number (> 1 million) of small molecules as SMILES [46–48] or “simplified molecular-input line-entry system”. In SMILES format, the information of a small molecule is reduced to a single-line ASCII string of characters [47]. This contains atoms in English letters, while bonding and stereochemistry are expressed by using special characters. Using a set of home-built scripts that utilize Open Babel commands, each of these strings was converted to three-dimensional structural forms. Finally, AutoDockFR was used to store them in a docking-ready protein data bank with partial charges or PDBQ format.

In parallel, protein structures were downloaded from PDB databank. The receptor and ligand molecules were separated using VMD. Hydrogens were added to both receptor and ligand molecules using AutoDockFR before storing them in PDBQ format.

### Molecular docking

High-throughput docking was accomplished by shellcodes that can parallelize a number of jobs using the GNU-parallel tool [49] across multiple nodes. The binding affinity is usually expressed quantitatively by the Gibbs’ free energy of binding (Eq. 1):

Eq. 1, $$\Delta_{\text{bind}}G^{o}(aq)=G\;(target\;\cdots \;ligand,aq)-G\;(target,aq)-G(ligand,aq)$$

where G(target⋯ligand,aq), G(target,aq), and G(ligand,aq) are defined as the aqueous-state Gibbs’ free energies of the ligand-bound target, the free target, and the free ligand, respectively. The $\Delta_{bind}G^{o}(aq)$ values, obtained from the docking output, were randomly separated into three train-validation-test groups with 70, 15, and 15 percentage splits, respectively. The greater binding affinity corresponds to more negative $\Delta_{bind}G^{o}(aq)$ values. A threshold of $\Delta_{bind}G^{o}(aq)$ was calculated based on the statistical representation of the training set data for a specific target active site (*vide infra*).

### Convolutional neural network (CNN) fingerprint

The convolutional neural network (abbreviated hereafter as CNN) fingerprints were generated by modifying Morgan’s algorithm [50], which produces extended connectivity fingerprints (ECFP) [51]. In the original algorithm, first generated by Morgan in 1965 to solve molecular isomorphism problem, each atom of a molecule was represented as graph, with atoms as vertices and bonds as edges. First, a terminal atom of a molecule is chosen, and using atom type and bonding information, a unique identifier number is assigned to the first vertex. Next, following Daylight atomic invariants rule [51], a given radius around the chosen terminal atom, adjacent graph vertices (i.e. other atoms) are identified, and a sub-structure is produced, in which a sum of adjacent graph vertices (with atom and bond information) is converted to a unique number identifier using a hash function. The sphere is then increased in the next cycle to include the next layer of atoms. When this procedure converges with all atoms considered, each identifier is converted to a bit array to generate binary machine-readable form.

In the present study, instead of a fixed ECFP [51] as input, a CNN-based fingerprint-generation was implemented [52], which involves graph-based convolutions to carry out weight and bias optimizations within the neural network architecture (Scheme 1).

An algorithm was developed to transform the information about the molecule into a graph structure, where atoms represent nodes and bonds represent edges. The molecular graph is defined using 3 matrices, a matrix representing specific features of each atom in the molecule, a matrix representing specific features of each bond in the molecule, and a matrix representing the connectivity of atoms and bonds in the form of a graph (Scheme 1). This last matrix is very similar to a simple connectivity matrix, with extra information characterizing the unique bond, which connects any two atoms. These matrices represent the input into the overall machine learning structure, specifically the convolutional model which generates the neural fingerprint, an optimized, trainable analog to general chemical fingerprints like the ECFP.

In the convolutional model, at each layer, a one-dimensional fingerprint of a specified length was created, which is a real-valued vector. The process of creating these fingerprints was similar to the generation of ECFP. However, differentiable functions replaced non-differentiable functions in the generative algorithm, in turn allowing the inclusion of trainable weights and biases in this process, which were optimized during the training process. Some examples include the non-differentiable hash and array-indexing functions that can be replaced by the continuous and differentiable sigmoid and softmax functions. The fingerprints at each convolutional layer are connected just like an artificial neural network (ANN). Typically, such a model is still called convolutional, as the generation of the fingerprints involves the combination of information from the three matrices consisting of atom features, bond features, and the molecular graph. These three layers of information is embedded into these fingerprints in a usable manner. The last layer produces the final fingerprint which is then mapped to the ANN (Scheme 1). Note that the trainable weights and biases in the convolutional fingerprint generation process were not trained separately to the ANN trainable parameters. The backpropagation of the loss from the binary ANN output included the trainable weights and parameters included in the generation of the fingerprint. Hence, this enabled the fingerprints from molecules to be optimized based on the task presented to the model, which in the present scenario is predicting binding affinity of a certain active site of a protein target.

### Artificial neural network (ANN)-based binary classification

This ANN is a binary classification model based on a calculated binding affinity threshold. To obtain the best set of hyperparameters in training and evaluating the model, the optimizer developed by Adams *et al*. [53] was used. A hyperparameter search was carried out through a simple grid search technique with unique combinations of the following hyperparameters: weight decay, learning rate, dropout frequency, batch size, and fingerprint length. Custom initialization was used in regard to both the ANN and fingerprint weights and biases. Fingerprint weights were initialized based on a normal distribution of the Xavier initialization method [15], which was shown to be favorable for the sigmoid activation function. Bias was initialized to a constant 0.01 for both the ANN and fingerprint. At each convolutional layer (including the initial input layer), an output layer transforms the associated atom features into a real-valued vector: the fingerprint. The vector sum of these intermediate fingerprints resulted in the final fingerprint, which was mapped to the first layer of a simple Artificial Neural Network (ANN).

### Oversampling to induce a balanced dataset

In a statistically unbiased sample, binary classes comprise 50% of the dataset, enabling the model to be trained on wider features that defines each class. However, this is not true for the present case. The docking data exhibited that weakly-bound molecules greatly outnumber those exhibiting tighter binding. Thus, to test the predictive capabilities, the threshold $\Delta_{bind}G^{o}(aq)$ was determined such that a varying pre-defined percentage of molecules would be considered as “hits” or a “1” in terms of binary classification. This measure resulted in only the top 5%, 10%, or even 20% of molecules as hits. As a countermeasure, oversampling was enforced on the training dataset by randomly duplicating the fingerprints of hits such that the distribution of hits and non-hits was balanced at 50% for each class. The validation and test datasets were not altered in any way.

### Evaluation metrics

All metrics presented were solely used to quantify results for the neural network architecture’s final predictive capabilities on the test dataset. Precision is a general metric that quantifies the proportion of hits predicted by the neural network that were true hits based on their $\Delta_{bind}G^{o}(aq)$ values as in Eq. 2

Eq. 2, $$precision=\frac{TP}{\left(TP+FP\right)}$$

where true positives (TP) refer to the number of predicted hits that were true hits and false positives (FP) refer to the number of nonhits that were predicted as hits. Recall is another metric that goes hand-in-hand with precision and relays the proportion of actual hits that were predicted as hits by the neural network (Eq. 3)

Eq. 3, $$recall=\frac{TP}{\left(TP+FN\right)}$$

where false negative (FN) refers to the hits that were predicted as non-hits by the neural network. The receiver operating characteristic (ROC) and area under the curve (AUC) is a metric often used in binary classification problems. The ROC curve plots the *true positive rate* against the *false positive rate* at different classification thresholds. The AUC, ranging from 0 to 1, is then called the ROC AUC. A higher AUC generally indicates better performance at binary classification.

The area under the precision-recall curve or PR-AUC plots the precision and recall at different probability thresholds to visualize the tradeoff between the two at a given probability threshold. The area ranges from 0 to 1 and, generally, higher values indicate better, more consistent precision and recall for a model.

The predictive enrichment probability (PEP) is expressed as the probability of the model predicting a molecule as a hit given that the molecule is really a hit. In particular, this probability ranges over the $\Delta_{bind}G^{o}(aq)$ values that are lower than the threshold defined prior to training the model. In theory, this metric indicates how well a model can capture binary hits over a continuous distribution of values.


Eq. 4 $$PEP=\frac{\mathrm{Numberofmoleculespredictedasahit}|\Delta_{\text{bind}}G^{\circ }(aq)<\text{threshold}}{\mathrm{Totalnumberofmolecules}|\Delta_{\text{bind}}G^{\circ }(aq)<\text{threshold}}$$

Lastly, the $F_{1}$ score is defined by the harmonic mean of the precision and recall as in Eq. 5.


Eq. 5 $$F_{1}=\frac{2}{\left[(\mathrm{precision}{)}^{-1}+(\mathrm{recall}{)}^{-1}\right]}$$

## Results and Discussion

### Enzyme targets

**Figure 1:**
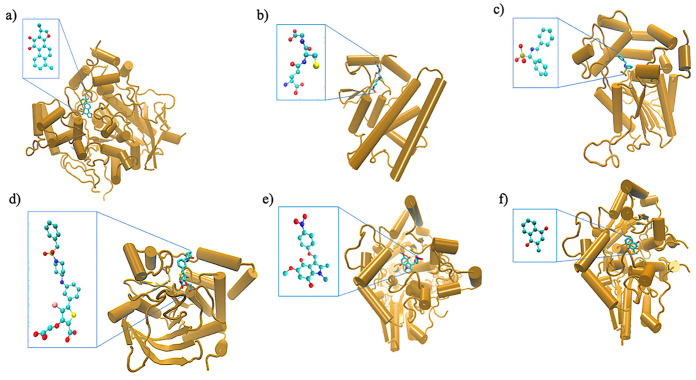
The folds of the enzymes and the active site-bound small molecules (shown in the insets) for a) ACEase with dihydrotanshinone I, b) GLTase with glutathione, c) PAPase with a-benzyl-aminobenzyl-phosphonic acid, d) PTPase with ligand ID: 527, e) QR1ase with ligand ID: ES936, and f) QR2ase with menadione.

In this study, six distinct enzymes were chosen that are well regarded as potential therapeutic targets for various diseases. The following is a concise description of the structure-function of these enzymes related to their recognition as drug targets. As shown in Fig. 1, these enzymes have different folds, and the bound small molecules are diverse in terms of their structures.

#### Acetylcholinesterase.

Abbreviated as ACEase, hereafter, is responsible for the regulation of neurotransmission through the degradation of acetylcholine (the neurotransmitter) in synapses of the nervous system [54]. Inhibitors of this enzyme are sought as they can be used as therapeutics for treatment of disease and protection against nerve agents. The X-ray crystal structure representing the inhibitor dihydrotanshinone I-bound target (PDB code 4m0e [54]) was used for this study.

#### Glutathione S-transferase.

Abbreviated hereafter as GLTase [55], is responsible for adding electrophilic group to glutathione (the tripeptide formed with cysteine, glycine, and glutamic acid) and is responsible for detoxification. Additionally, they are involved in promoting tumor pathogenicity and chemoresistance [56]. The current study was based on the protein structure (PDB code: 1pkw [55]) in complex with glutathione (Fig. 1b).

#### Prostatic acid phosphatase.

The prostatic acid phosphatase enzyme (abbreviated hereafter as PAPase) is responsible for the malignant growth of cells [57]. Inhibitors of this enzyme can be used as therapeutics for prostate cancer. The enzyme (PDB code: 1nd5 [57]) falls into the subclass of protein tyrosine phosphatase and is responsible for the dephosphorylation of epidermal growth factor receptor. The a-benzyl-aminobenzyl-phosphonic acid-bound structure is shown in Fig. 1c.

#### Protein tyrosine phosphatase 1b.

Also belonging to the class of protein tyrosine phosphatase, this enzyme (abbreviated as PTPase) regulates negatively insulin [58]. This enzyme is an attractive target for type 2 diabetes and obesity and the drug (5-(3-{[1-(benzylsulfonyl) piperidin-4-yl]amino}phenyl)- 4-bromo-3-(carboxymethoxy) thiophene-2-carboxylic acid; ligand id: 527)-bound X-ray crystal structure used in the study belongs to the PDB code: 2qbp [58].

#### NAD(P)H:quinone oxidoreductase type 1.

This oxidoreductase enzyme is responsible for protecting cells against cellular toxicity due to free radicals [59]. This enzyme (abbreviated as QR1ase) is overproduced in cancerous cells and therefore selective inhibitors of the enzyme have strong chemotherapeutic potentials. The present study is carried out on the 5-methoxy-1,2-dimethyl-3-(4-nitrophenoxymethyl)indole-4,7-dione (ligand ID: ES936)-bound X-ray crystal structure (Fig. 1e) of the enzyme bearing the PDB code: 1kbq [59].

#### NRH:quinone oxidoreductase.

This is another oxidoreductase enzyme (abbreviated as QR2ase) involved in regulating cellular toxicity and oxidative stress [60]. The enzyme is targeted for anti-Alzheimer disease drug development. As illustrated in Fig. 1f, the X-ray crystal structure of the menadione-bound enzyme (PDB code: 2qr2) was used in the study [60].

### Parameters and metrics for benchmarking

Based on the essence of compound screening, the present model was optimized to retain molecules with relatively higher binding affinities while ‘filtering’ as many molecules with relatively lower binding affinities. Therefore, the use of larger, preliminary datasets of small molecules would become feasible. Using various metrics to benchmark, the performance of models was compared, both between the same architecture and between different architectures, e.g. convolutional fingerprint and Morgan fingerprint models. For a general metric, AUC measured the quality of the models’ predictions across different classification thresholds. To demonstrate how well these “filtration” architecture performs, increased significance was placed on the true positive rate (sensitivity). The purpose of compound screening is to retain the ligands that most favorably bind to a receptor. In turn, it is increasingly important that the number of false negatives was minimized in order to not discard any potentially promising compounds. Hence, sensitivity is maximized as much as possible without handicapping the model’s ability to be precise in its predictions.

#### Receiver operative characteristic and precision-recall curves.

**Figure 2:**
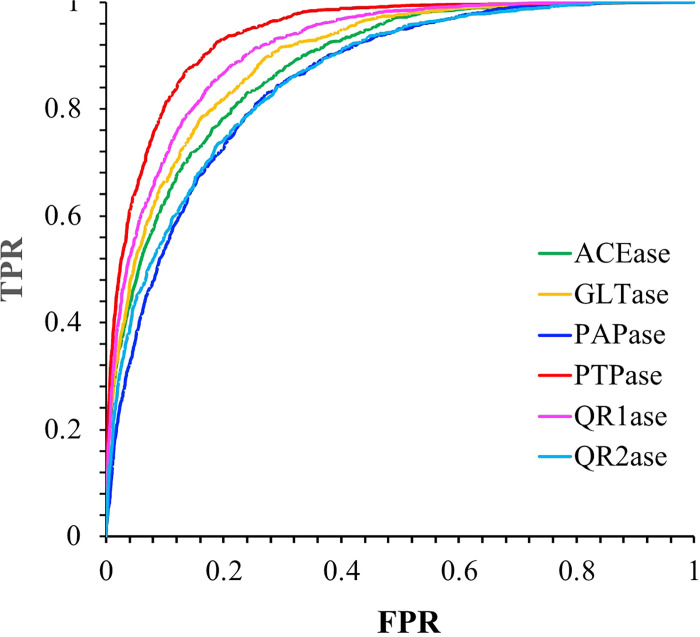
The receiver operative characteristic (ROC) curves for the six models, namely ACEase, GLTase, PAPase, PTPase, QR1ase and QR2ase. The AUC values are given in Table 1.

The results consisting of receiver operative characteristic (ROC) curves, precision-recall (PR) curve, are presented (Table 1). These results are based on a hyperparameter grid search undertaken only a single time to ensure a notion of randomness in the quantitative performance across all proteins.

across all proteins. Results described below are for single models chosen from the hyperparameter grid search. Parameters considered when choosing this ‘best model’ include precision, recall, and average $\Delta_{bind}G^{o}(aq)$ of the predicted hits. The ROC curves of the six models (Fig. 2), with respective AUC values in Table 1.

**Figure 3:**
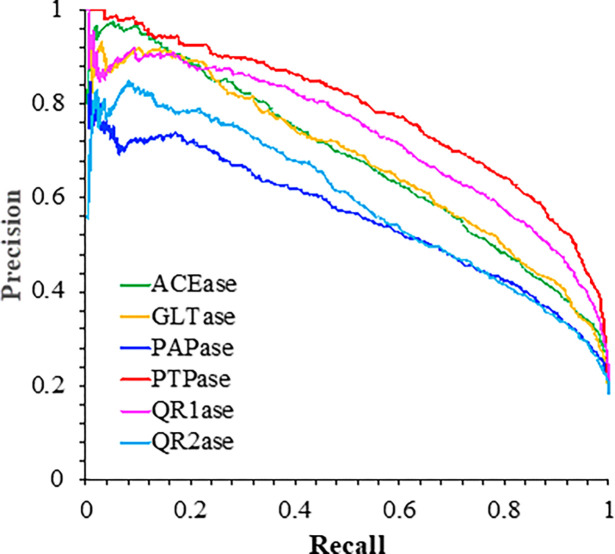
The precision-recall (PR) curves for the six models, namely ACEase, GLTase, PAPase, PTPase, QR1ase and QR2ase. These curves are plotted based on the best selected model for each protein alongside corresponding PR-AUC values given in Table 1.

Moreover, the precision-recall curve (Fig. 3) with corresponding AUC values have also been included in order to gauge the tradeoff between precision and recall along various thresholds. Of course, these models were constructed such that recall is maximized (Fig. 3). However, in order to efficiently ‘filter’ out molecules with higher negative $\Delta_{bind}G^{o}(aq)$ values, it is important for these models to be able to also classify the said molecules effectively.

These fundamental binary classification parameters relay two important things: the nature of the imbalanced dataset as well as the suitability of the model based on the protein being investigated. Both the ROC-AUC and PR-AUC values vary based on the protein. As a result, it is evident that the neural network architecture was able to capture the significant affinity (i.e. large negative $\Delta_{bind}G^{o}(aq)$ values) for molecular substructures at a higher level for proteins such as PTPase compared to PAPase. Moreover, compared to other deep-learning-based studies,[16, 17] the ROC-AUC values seem to be more consistent across the wide array of proteins selected for this study. Although specific metrics may vary, this observation seems to substantiate the classifier’s ability to perform well at different thresholds for many different proteins.

#### Predictive enrichment probability.

**Figure 4.**
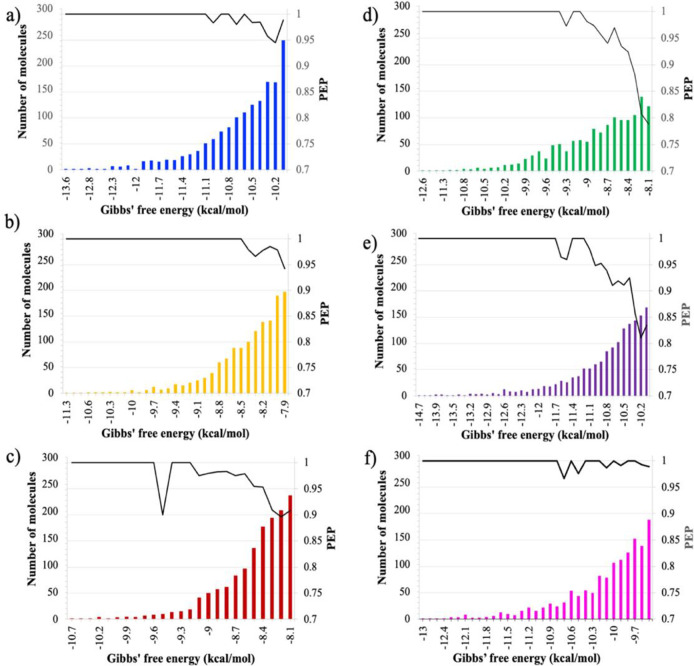
Predictive enrichment probability graphs and the total number of molecules with which the PEP was calculated over the range of $\Delta_{bind}G^{o}(aq)$ values in the binary ‘hit’ range. Note that the counts displayed as bar graphs are not the number of correctly predicted molecules but rather the total number of molecules in the test dataset at each $\Delta_{bind}G^{o}(aq)$ value.

To further visualize and quantify the models’ abilities in correctly predicting those molecules that have a lower $\Delta_{bind}G^{o}(aq)$ value than the predefined threshold (e.g. recall), the predictive enrichment probabilities or PEP is calculated (Eq. 4) based on the set of hits in the test dataset for each protein (Fig. 4). The PEP calculated at each Gibbs’ free energy value lower (or equal) to the Gibbs’ free energy threshold that was used in training. The granularity of the $\Delta_{bind}G^{o}(aq)$ values is found to be 0.01. Below, six graphs are shown illustrating the PEP alongside the number of molecules in the test dataset that are hits at each Gibbs’ free energy value.

The PEPs for all proteins indicate that the neural network architecture is able to firmly grasp the molecular substructures that correlate to a lower $\Delta_{bind}G^{o}(aq)$ value. Particularly, those molecules with extremely low $\Delta_{bind}G^{o}(aq)$ values (Fig. 4, towards the left of the graphs) are almost always predicted correctly as hits by the neural network architecture. This is imperative in a good docking-based machine-learning model. It is necessary to filter out as many molecules while incurring the least amount of loss of tightly-bound molecules to the protein, which in this case means the loss of molecules with very low $\Delta_{bind}G^{o}(aq)$ values. Without this characteristic, it would become increasingly infeasible to use such models in lieu of traditional docking techniques.

#### Metrics for evaluation.

The pertinent parameters of all models using the neural network architecture presented in Table 2. All parameters are calculated based on final predictions using the test dataset after training and validation.

It is clear to see the effect of an imbalanced dataset on the results based on the F_1_ score (Eq. 5) and ROC-AUC values (Table 2). Although recall is greatly maximized, precision remains relatively low. Although that is the purpose of the architecture, there may be further optimizations that can be implemented in regard to the architecture to enable the model to more performantly discard molecules with lower $\Delta_{bind}G^{o}(aq)$ values. Nevertheless, the extremely high recall attests to the architecture’s ability to retain molecules that favorably bind to the protein being investigated. Moreover, some proteins seem more favorable in investigating using this model than others (as was mentioned previously). In the case of PTPase, around 93% of molecules in the bottom 20% of $\Delta_{bind}G^{o}(aq)$ values are retained whereas, based on the relatively high precision value, a good chunk of the overall dataset seems to be reduced. All in all, the architecture quantitatively performs very well at retaining molecules meaningfully.

#### Comparison of similarity maps.

**Figure 5:**
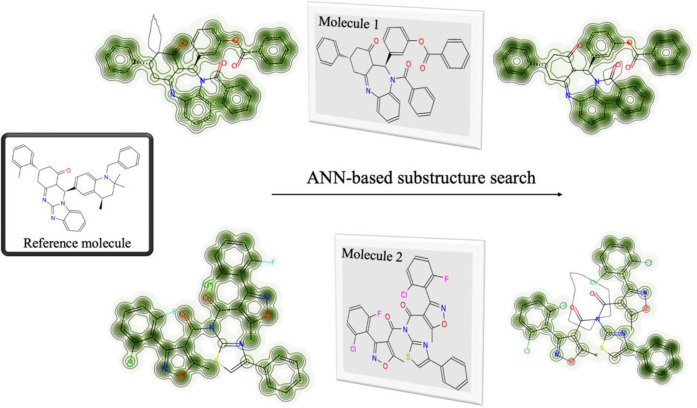
A visual assessment of the superiority of the neural fingerprint-based model in predicting substructure similarity between molecules. The similarity maps were calculated using random forest classification. The darker green contour lines indicate greater similarity, and the red atoms indicate dissimilarity.

This was visually assessed by comparing the similarity maps[43] of substructures. Figure 5 illustrates the visual comparison of two molecules (top and bottom), whose similarity to the reference molecule (in the left) was assessed using two datasets: one used in the neural fingerprint-based model versus the regular docked data. As demonstrated in the figure, the neural fingerprint-based model represents a superior discrimination with the darker lines indicating greater similarities in the top molecule. In contrast, the bottom molecule represents larger dissimilarity in neural fingerprint-based training compared to the dataset obtained from the docking score alone (Fig. 5).

#### Comparison with Morgan fingerprints.

The effectiveness of the neural fingerprint-based architecture for compound screening was compared to the commonly-used Morgan fingerprint-based[50] machine learning models. Specifically, the model and parameters used for the Morgan fingerprint-based “deep docking” study reported by Gentile et. al.[16, 17] were included in the comparative analysis. The “deep docking” algorithm used an iterative docking protocol, but the present analysis is carried out using a single iteration of this protocol. Both models calculated the threshold values to transform a continuous distribution of molecules over $\Delta_{bind}G^{o}(aq)$ values into a binary classification problem. The models were compared with the same protein, ACEase, on the same overall dataset of molecules, and with the same threshold of − 10.0 kcal/mol. This value corresponded to around an 80 – 20 ratio of non-hits and hits in the dataset. Another hyperparameter grid search and training session was conducted for this comparison in regard to the neural fingerprint architecture (Table 3).

The precision and recall values (Table 3) seem to characterize both models appropriately. The Morgan fingerprint-based “deep docking” architecture tries to balance the two parameters as much as possible, whereas the neural fingerprint model maximizes the recall. However, combing through the results of all models trained for the “deep docking” architecture, it is apparent that the range of precision and recall seems to remain relatively constant despite changing hyperparameters (Table 3). In contrast, in the case of the neural fingerprint model, there exists significant variability in the values of these metrics. For example, while there are models that maximize recall like the one from which the results above are derived, there are also others that balance the precision and recall similarly to the Morgan fingerprint model (data not shown). One such model has a recall of 0.74 and a precision of 0.55. There are also models at the other extreme, which try to capture all the hits in the dataset without regard to a high level of precision (recall = 0.99, precision = 0.28). This represents the ability of the neural fingerprint architecture to adequately present users with the option of training for the filtering of ‘bad’ molecules, the maintenance of ‘good’ molecules, or somewhere in between.

In summary, these results demonstrated the plasticity of the neural fingerprint-based docking model and its significant ability to capture hits. Even using a smaller dataset, the algorithm was able to map chemical space with adequate efficacy.

## Conclusions

The perpetual increase of drug design-relevant candidates in easily accessible databases has certainly enriched prospects of identifying drug molecules that can selectively inhibit a specific target. Still, though, work in optimizing workflows and expediting methods for compound screening has not been able to keep up with this increase in indexed small molecules. Thus, using convolutional neural network-derived fingerprints a machine learning-based drug screening technique has been generated for faster compound screening. The architecture takes in, as input, various vectors that describe the structure of a molecule in terms of a graph. A differentiable, neural network-based fingerprint was calculated, and mapped to an Artificial Neural Network which then outputs a continuous value and is interpreted based on a binary classification problem.

It was shown that the model performs extremely well at retaining molecules that are classified as favorably binding to a specific protein. Based on the observed recall, ROC-AUC, and PR-AUC, the model performs reasonably well over a range of drug targeted proteins. Compared to previous works, the ability of this architecture to maximally retain favorable molecules is greater. However, various trained models present various characteristics, with some maximizing recall, maximizing precision, or finding a balance between both parameters. Hence, users of this model are enabled to choose models generated through the hyperparameter search based on use or circumstance. This demonstrates the plasticity of the neural fingerprint-based screening architecture. Furthermore, the relatively small working set of 50000 molecules utilized as the overall dataset in this work illustrates the architecture’s ability to learn without the hundreds of thousands or millions of molecules required in previous works. The lower precision values may not be suitable if a small, final working set of molecules is needed. Of course, the choice remains with the user based on the range of model priorities previously discussed. However, if the retention of favorably binding molecules was crucial alongside a small final dataset, this protocol would be necessary to use. In this case, an iterative protocol, such as that used and developed by Gentile et. al. [16, 17] would be the best option. By iteratively reducing the dataset with smaller and smaller datasets, the Gibbs’ Free Energy would continue to fall until only molecules. This necessitates experimentation; however, this would be a good first step in developing a protocol using the architecture presented in this work.

In summary, this model offers labs and organizations the ability to conduct virtual, molecular screening without a lot of resources in terms of cost and time inherent with the traditional processes. The architecture presented in the current study provides a practical tool for screening variable-sized molecular databases. The novelty of the present algorithm lies in the speed of the training and tuning of its models before testing and validating its predictive capabilities. Additionally, this model is capable of mapping similar chemical spaces by using a smaller dataset of molecules as compared to contemporary algorithms and hence can be considered as a significant step forward in the field of machine learning-embedded computational drug discovery.

## Figures and Tables

**Table 1:** Relevant metrics for benchmarking obtained in the study.

Enzyme system/ Metrics	AUC	PR-AUC
ACEase	0.88	0.68
GLTase	0.90	0.68
PAPase	0.85	0.56
PTPase	0.94	0.78
QR1ase	0.91	0.73
QR2ase	0.86	0.59

**Table 2:** The recall, precision, ROC-AUC, PR-AUC, and F1 score for the six protein systems studied. All values are calculated based on model predictions on the test dataset after training.

Enzyme system/ Metrics	Recall	Precision	ROC-AUC	PR-AUC	F1 Score
ACEase	0.98	0.30	0.88	0.68	0.47
GLTase	0.98	0.29	0.90	0.68	0.45
PAPase	0.94	0.31	0.85	0.56	0.47
PTPase	0.93	0.50	0.94	0.78	0.65
QR1ase	0.92	0.47	0.91	0.73	0.62
QR2ase	1.00	0.22	0.86	0.59	0.36

**Table 3:** Comparison of Precision, Recall, and Area Under the ROC Curve between both architectures. All valueswere calculated using the scikit-learn library and based on model predictions on randomly-sampled testdatasets.

Metrics	Neural fingerprint architecture	Morgan fingerprint architecture (Gentile *et. al.*[16, 17])
Precision	0.40	0.58
Recall	0.92	0.70
ROC-AUC	0.90	0.90

## Data Availability

The codes developed for neural network-based models are available in https://github.com/rivmons/nfp-docking.
